# Development and initial validation of the Engagement in Athletic Training Scale

**DOI:** 10.3389/fpsyg.2024.1402065

**Published:** 2024-07-23

**Authors:** Jing-Dong Liu, Jun-Xia Wu, Yu-Duo Zou, Zhong-Hao Wang, Shuge Zhang, Jin-Chuan Hu, Hao Liu

**Affiliations:** ^1^Department of Physical Education, Sun Yat-sen University, Guangzhou, China; ^2^Hebei Sports University, Shijiazhuang, China; ^3^Organization Department of the CPC Jinzhou Municipal Committee, Jinzhou, China; ^4^University of Derby, Derby, United Kingdom; ^5^School of Physical Education, Kunming University of Science and Technology, Kunming, China; ^6^Physical Education School, Shenzhen University, Shenzhen, China

**Keywords:** athlete engagement, engagement in athletic training, validity, reliability, measurement invariance

## Abstract

The current study presents the development process and initial validation of the Engagement in Athletic Training Scale (EATS), which was designed to evaluate athletes’ engagement in athletic training. In study 1, item generation and initial content validity of the EATS were achieved. In study 2, the factor structure of the EATS was examined using exploratory factor analysis (EFA) and exploratory structural equation modeling (ESEM). Internal consistency reliabilities of the subscales were examined (*N* = 460). In study 3, factor structure, discriminant validity, internal consistency reliability, and nomological validity of the EATS were further examined in an independent sample (*N* = 513). Meanwhile, measurement invariance of the EATS across samples (study 2 and study 3) and genders was evaluated. Overall, results from the 3 rigorous studies provided initial psychometric evidence for the 19-item EATS and suggested that the EATS could be used as a valid and reliable measure to evaluate athletes’ engagement in athletic training.

## Introduction

In competitive sports, athlete burnout has been extensively studied because it may result in profound negative consequences, including mental health problems, poor performance, injuries, and dropout of sports (Isoard-Gautheur et al., 2012; Gustafsson et al., 2017; Martinent et al., 2020). Researchers and practitioners have invested significant resources and efforts to better understand the mechanisms of burnout development and to prevent the onset of burnout symptoms (Raedeke and Smith, 2001). With the spread of positive psychology, which emphasizes human resources and potentials, rather than malfunctioning and weakness (Seligman and Csikszentmihalyi, 2000), researchers in sports fields started paying attention to the engagement of athletes. These studies advocate that the promotion of positive sports experiences of athletes, rather than the treatment and prevention of malfunctioning and negative experiences of athletes, should be given more attention. Lonsdale et al. (2007b) conducted a series of four studies to qualitatively conceptualize the structure of athlete engagement (AE) and quantitatively develop and validate a psychometric sound instrument measuring athlete engagement (Lonsdale et al., 2007a). The Athlete Engagement Questionnaire (AEQ) includes 16 items measuring 4 dimensions of dedication, confidence, vigor, and enthusiasm. Athletes used a 5-point Likert scale (1 = almost never; 2 = rarely; 3 = sometimes; 4 = frequently; 5 = almost always) to indicate “how often they felt this way in the past four months.” Dedication represents a desire to invest effort and time toward achieving goals one views as important (e.g., I am dedicated to achieving my goals in sports). Confidence refers to a belief in one’s ability to attain a high level of performance and achieve desired goals (e.g., I am confident in my abilities). Vigor is defined as physical, mental, and emotional energy or liveliness (e.g., I feel energized when I participate in my sport). Enthusiasm reflects the feelings of excitement and high levels of enjoyment (e.g., I feel excited about my sport). Since the development of the AEQ, it has been widely used in athlete engagement studies and translated into different languages (Martins et al., 2014; Wang et al., 2014; De Francisco et al., 2018). Conceptualization of athlete engagement and the development of the AEQ allow researchers to better understand and promote positive sports experiences of athletes (Lonsdale et al., 2007b; Hodge et al., 2009).

It is noteworthy that the initial research on athlete engagement in competitive sports was mainly inspired by studies on work engagement (Schaufeli et al., 2002). Lonsdale et al. borrowed the concepts and structure of work engagement to conceptualize and construct athlete engagement (Lonsdale et al., 2007a,b). Schaufeli et al. (2002) defined work engagement as a positive, fulfilling, work-related state of mind that was characterized by vigor, dedication, and absorption. Following a similar logic, Lonsdale et al. (2007b) revealed three core AE dimensions of confidence, vigor, and dedication in their qualitative study with confidence being considered as the context-specific dimension. Therefore, athlete engagement was initially defined as a persistent, positive, cognitive-affective experience in sports that was characterized by confidence, dedication, and vigor (Lonsdale et al., 2007b). In their further quantitative studies, the enthusiasm dimension was added because it was differentiated from the vigor dimension and finally formed the four-dimensional construct of the AEQ based on the results of sequential statistical analyses (Lonsdale et al., 2007a). Kahn, the pioneering researcher on employee engagement, proposes that engaged employees should be physically, emotionally, and cognitively involved in their work roles, which implies that employee engagement may include physical, cognitive, and emotional components (Kahn, 1990). Similarly, in the academic setting, researchers have reached a consensus that the construct of engagement is multidimensional and includes behavioral, cognitive, and affective (or emotional) aspects, operating collectively to reflect individuals’ positive approach to learning activities (Nystrand and Gamoran, 1991; Fredricks et al., 2004; Appleton et al., 2008; Alrashidi et al., 2016). Although Lonsdale et al. (2007a) claimed to include cognitive and affective components of AE in their definition, this differs somehow from the assumptions on work engagement by Kahn (1990) and the engagement in academic settings (Fredricks et al., 2004; Appleton et al., 2008). Specifically, the dedication, absorption, and vigor in Schaufeli et al. (2002) and confidence and enthusiasm in Lonsdale et al. (2007a) describe individuals’ psychological engagement rather than their behaviors (Schaufeli et al., 2002; Upadyaya and Salmela-Aro, 2013). As a result, Schaufeli et al.’s (2002) construct of engagement was considered missing information pertaining to individuals’ attendance, adherence to social norms, and following the rules (Upadyaya and Salmela-Aro, 2013; Alrashidi et al., 2016). Therefore, athlete engagement is mainly related to the continuous and positive emotional and cognitive feelings of athletes toward their sports, which does contribute to the quality of training, but cannot directly reflect to what extent athletes engaged in specific sessions of their athletic training, especially at behavioral level. Athletic training involves various behavioral and cognitive activities guided by particular requirements (e.g., tactical and technical skills; training load and intensity), which dynamically influence and are influenced by athletes’ affective states in the process of performing these activities (Gabbett et al., 2017; Neupert et al., 2024). Therefore, engagement in athletic training (EAT) is different from athlete engagement but similar to academic engagement, which requires individuals to behaviorally, cognitively, and affectively engage in their learning and study activities (Nystrand and Gamoran, 1991). Ben-Eliyahu et al. (2018) defined engagement as the intensity of productive involvement with an activity, encompassing three distinctive yet related components: behavioral, affective, and cognitive engagement. There are some typical scenarios often observed in academic settings where students could be emotionally engaged in tasks at hand but not thinking about the learning at all (Appleton et al., 2008). Some students could be thinking about materials and experiencing emotions but not implementing any learning activities at the behavioral level (Reschly and Christenson, 2022). Some students could be behaviorally active but not cognitively and affectively engaged in the task at hand (Appleton et al., 2006). Similar scenarios are also often observed in athletic training settings. For example, athletes may be physically repeating the movements and actions but without thinking about the reasons and requirements of the training tasks at all (Kuokkanen et al., 2021; Montull et al., 2022). Some athletes may think too much about the techniques and worry about making mistakes, especially under pressure from coaches, leading them to be reluctant to try out physically (Kuokkanen et al., 2021). These kinds of scenarios will be harmful to the quality of athletic training and could not be reflected by athlete engagement.

Athletic training aims at improving and maintaining competitive competence and sports performance of athletes, which is a planned sports activity that is led and guided by coaches and mainly engaged by athletes. Therefore, scientific and reasonable plans and contents (tasks, number of repetitions, intensity and volume, etc.) arranged by coaches in athletic training are the basis of the training quality while the extent to which athletes engage in these training tasks is the guarantee of the training quality. Previous research has extensively evaluated training quality from physical, technical, and tactical perspectives (Saw et al., 2016; Jeffries et al., 2020; Inoue et al., 2022; Bucher Sandbakk et al., 2023; Shell et al., 2023); no research has investigated how athletes’ engagement in athletic training may contribute to their training quality. The main reason derives from two unsolved questions: (1) the concept and structure of the athletes’ engagement in athletic training are not clarified yet; (2) no valid and reliable instrument could be used to assess athletes’ engagement in athletic training. Although Reynders et al. (2019) adjusted academic engagement items to the sports context to assess athletes’ behavioral engagement, affective engagement, and cognitive engagement by changing terms, such as “teacher” into “coach” and “learning-related activities” to “training or competition,” they did not differentiate the athletic training from competitions, which are two different settings with context-specific characteristics. In addition, only internal consistency reliability but other psychometric properties of the modified instrument were evaluated and reported in Reynders et al. (2019) study, which leaves an open question that not sure whether the modified instrument from an academic setting could be used to assess athletes’ engagement in athletic training. Collectively, it is imperative to clarify the concept and structure of engagement in athletic training and further develop a psychometrically sound and context-specific instrument that could be used to assess athletes’ engagement in athletic training.

To shed light on these two questions, three studies were organized. In study 1, the concept and structure of the engagement in athletic training were clarified and the item pool of the Engagement in Athletic Training Scale (EATS) was generated using a qualitative approach. In study 2, factor structure, discriminant validity, and internal consistency reliability of the EATS were initially evaluated in a sample of Chinese athletes. In study 3, factor structure, discriminant validity, internal consistency reliability, and nomological validity of the EATS were further examined in an independent sample of Chinese athletes. Meanwhile, measurement invariance of the EATS across samples and genders was evaluated. Ethical approval for the entire project (involving three studies) was obtained from a local university’s human and animal research ethics committee (Reference No. M202300127).

## Study 1: item generation and content validity of the EATS

The purpose of study 1 was to develop a preliminary version of the EATS and further evaluate its content validity. First, based on previous research on work engagement and student engagement, athletes and coaches were interviewed to explore the definition and structure of engagement in athletic training. Second, the pool of EATS items was developed based on interviews with athletes. Third, the content validity of the preliminary version of the EATS was evaluated by athletes, coaches, and sports psychologists.

### Method of study 1

#### Participants

##### Participants for interview

Six coaches (two women) and nine athletes (five women) were invited to participate in a series of face-to-face semi-structured interviews. All participants were competing at national or international games at the moment of data collection. Basic information of participants is presented in Table 1.

**Table 1:** Basic information of participants in three studies.

	Participants	Sample	*N* (female)	Age M (SD), Range	Professional working/Training experiences M (SD), Range	Sports/services for sports
Study 1	Interview	Athletes	9 (5)	21.67 (5.00), 14–28	8.78 (3.63), 4–15	fencing, volleyball, rhythmic gymnastics, and swimming
		Coaches	6 (2)	41.33 (7.15), 34–50	15.67 (7.5), 8–25	fencing, martial arts, shooting, taekwondo, and rhythmic gymnastics
	Content validity	Athlete sample 1	16 (9)	19.94 (2.64), 15–23	6.75 (1.34), 4–9	boxing, taekwondo, judo, gymnastics, track and field, and fencing
		Athlete sample 2	22 (12)	20.32 (2.45), 16–24	6.32 (1.08), 4–8	boxing, taekwondo, swimming, badminton, martial arts, table tennis, gymnastics, rhythmic gymnastics, synchronized swimming, fencing, and volleyball
		Sport psychologists	7 (1)	43.28 (4.61), 35–50	11.85 (3.62), 7–18	fencing, swimming, judo, wrestling, diving, gymnastics, track and field, martial arts, tennis, rhythmic gymnastics, speed skating, and figure skating
		Coaches	16 (4)	43.18 (7.36), 30–55	13.37 (8.47), 4–30	boxing, fencing, soccer, basketball, swimming, taekwondo, rhythmic gymnastics, diving, beach volleyball, volleyball, and martial arts.
Study 2		Athletes	460 (221)	18.78 (3.76), 10–32	8.49 (3.89), 1–23	Track and field, swimming, water polo, diving, badminton, sanda, martial arts, table tennis, gymnastics, weight lifting, tennis, basketball, competitive trampoline, synchronized swimming, fencing, and volleyball
Study 3		Athletes	513 (271)	18.27 (3.95), 11–36	5.3 (4.18), 1–25	Track and field, table tennis, freestyle wrestling, weight lifting, basketball, baseball, softball, boxing, hockey, handball, and football

##### Participants for content validity

Sixteen athletes (athlete sample 1) were invited to evaluate whether the EATS items were clear and easy to understand. Twenty-two athletes (athlete sample 2) were invited to evaluate to what extent the EATS items were applicable to measure their EAT. All participants were competing at national or international games at the moment of data collection. Sixteen coaches were invited to evaluate to what extent the EATS items were applicable to evaluate athletes’ EAT in their coaching practice. All coaches had coaching experiences competing at national or international games. Seven sport psychologists were invited to evaluate to what extent the EATS items were applicable to measure athletes’ EAT. All sport psychologists had working experiences with athletes competing at national or international games. Basic information of participants is presented in Table 1.

#### Procedure

All participants were contacted through telephone or online to invite them to participate in this study. They were provided a copy of the invitation letter together with a written informed consent form, in which anonymity treatment was clarified and the research purpose of the study and requirements for participating in this study were clearly described. For interviews, coaches and athletes attended face-to-face semi-structured interviews, which happened in a quiet room and were audio-tapped (Supplementary material A: interview outline). Qualitative interviews were transcribed verbatim and analyzed using content analysis via NVivo 11 to clarify and identify the conceptual and operational definitions of athletes’ EAT and further generate a preliminary version of EATS (Supplementary material B).

For content validity evaluation, athlete sample 1 was invited to evaluate to what extent they can easily understand the EATS items using a 5-point Likert scale (1 = not clear at all, 5 = very clear). A response of 4 out of 5 from athletes suggests that they may have difficulties understanding the item, indicating that revision or removal of the item should be considered. Athletes (sample 2), coaches, and sports psychologists were invited to answer an online version of the EATS, in which the applicability of the EATS items in measuring athletes’ EAT was evaluated using a 5-point Likert scale (1 = inapplicable at all, 5 = highly applicable). They were also asked to provide suggestions for additional items if appropriate. The content validity index of each item (I-CVI) was computed by dividing the number of judges who gave a rating of 4 or 5 on a specific item by the number of judges (Lynn, 1986). In addition, modified kappa statistic (k^*^), an index of agreement of a certain type, was computed to further evaluate the content validity of items. The modified kappa statistic was proposed to address the issue of chance agreements for the CVI values by adjusting for chance agreements on the relevance of a certain type and excluding the chance agreements of any type (Polit et al., 2007). Therefore, it is a good supplementing indicator for the usage of CVI in content validity evaluation. According to Polit et al. (2007, p. 466), any I-CVI greater than 0.78 would indicate excellent, regardless of the number of judges. Guidelines described in Fleiss (1981) and Cicchetti and Sparrow (1981) were used as evaluation criteria for k^*^ (fair: *k* = 0.40–0.59; good: *k* = 0.60–0.74; excellent: *k* > 0.74). Both the I-CVI and k^*^ were subsequently used as a reference for deciding whether to retain, delete, or revise the item. It should be noted that the values of I-CVI and k^*^ converge with an increasing number of judges. With 10 or more judges, any I-CVI value greater than 0.75 yields a k^*^ greater than 0.75, which means that an I-CVI of 0.75 would be considered “excellent” with the panel consisting of more than 9 judges, but not with that of less than 9 judges (Polit et al., 2007). All participants participated in the study voluntarily and returned their consent form before the data collection. For athletes who were under 18 years old at the moment of data collection, informed consent was obtained from their coaches who were asked to act *in loco parentis*.

### Results of study 1

Being consistent with previous assumptions of engagement as a multidimensional construct, the results of the qualitative content analysis revealed that athletes’ EAT could be represented by three distinctive but correlated dimensions of behavioral engagement, cognitive engagement, and affective engagement. Athletes could be behaviorally active but not cognitively or affectively engaged in their training. For example, one could be actively repeating the training tasks but without thinking about why she or he should do it. Similarly, athletes may be intensively experiencing their affective feelings while not paying attention to coaches’ instructions or failing to achieve expectations from coaches. Logically, the three EAT components may influence each other and together affect their training quality. We defined the EAT as the intensity of athletes’ productive involvement with their training-related activities and tasks. The operational definition was organized as the extent to which athletes execute, comprehend, analyze, and affectively engage in their training-related tasks. Specifically, behavioral engagement refers to the extent to which elite athletes physically and behaviorally engage in their training activities including physical, skillful, and tactical-related training tasks. Cognitive engagement refers to the extent to which athletes correctly comprehend, actively analyze, and proactively concentrate on their training-related tasks. Affective engagement refers to the affective status that athletes express and experience throughout the training period including both positive and negative affect. According to the abovementioned conceptual and operational definitions, a preliminary version of the EATS was developed based on the qualitative content analyses, which includes 27 items with 10 items measuring behavioral engagement (example item: I successfully completed the training tasks assigned by my coach), 8 items measuring cognitive engagement (example item: I know exactly the purpose of my training tasks), and 9 items measuring affective engagement (example item: I enjoy the training process), respectively (Supplementary material B: 27-item EATS). Materials supporting the development of the definitions of EAT are available by request from the correspondence author.

It was found that athlete sample 1 reported an average score of 4.78 out of 5 ranging from 4.38 to 5 (the average percentage of scoring higher than 4 is 97% ranging from 88 to 100%), which means that the EATS items could be easily and clearly understood by athletes. Table 2 presents the I-CVI values and the modified kappa values (k^*^) computed-based responses of athletes, coaches, and sports psychologists. As the sample sizes of athletes (*n* = 22) and coaches (*n* = 16) were larger than 10, an I-CVI of 0.75 could be considered “excellent” (Polit et al., 2007). For the athlete sample, the I-CVI values of 6 items (item 1: 0.64; item 3: 0.45; item 7: 0.73; item 8: 0.55; item 16: 0.73 and item 20: 0.68) were lower than 0.75. The k^*^ values of the same 6 items were below 0.74 with 4 items (item 1: 0.64; item 7: 0.72; item 16: 0.72 and item 20: 0.67) falling between 0.60 and 0.74 (good) and 2 items (item 3: 0.35 and item 8: 0.47) lower than 0.59 (fair). These results suggested that 25 items were rated as good or excellent and 2 items (item 3 and item 8) were rated as fair. For the coach sample, the I-CVI values of 3 items (item 1: 0.63; item 3: 0.50; item 7: 0.69) were lower than 0.75. The k^*^ values of the same 3 items were below 0.74 with 1 item (item 7: 0.67) falling between 0.60 and 0.74 (good) and 2 items (item 1: 0.58 and item 3: 0.38) lower than 0.59 (fair). These results suggested that 25 items were rated as good or excellent and 2 items (item 1 and item 3) were rated as fair. For the sport psychologist sample, the I-CVI values of 3 items (item 3: 0.71; item 4: 0.57, and item 14: 0.57) were lower than 0.78. The k^*^ values of the same 3 items were below 0.74 with 1 item (item 3: 0.65) falling between 0.60 and 0.74 (good) and 2 items (item 4: 0.41 and item 14: 0.41) lower than 0.59 (fair). These results suggested that 25 items were rated as good or excellent and 2 items (item 4 and item 14) were rated as fair. No additional items were suggested by athletes, coaches, and sports psychologists. Given the inconsistent results observed across the three populations on the content validity of items, we decided to retain all items for the data collection in study 2. Removal or revision decisions would be made together with the results of the statistical analysis in study 2.

**Table 2:** The I-CVI values and modified kappa values.

Items	Athletes (*n* = 22)				Coaches (*n* = 16)				Sport Psychologists (*n* = 7)			
	M	I-CVI	p_c_	k^*^	M	I-CVI	p_c_	k^*^	M	I-CVI	p_c_	k^*^
1	4.05	0.64	0.01	0.64	4.06	0.63	0.12	0.58	4.57	1.00	0.00	1.00
2	4.50	1.00	0.00	1.00	4.44	0.94	0.00	0.94	4.29	0.86	0.05	0.85
3	3.32	0.45	0.15	0.35	3.25	0.50	0.20	0.38	4.14	0.71	0.16	0.65
4	4.36	0.91	0.00	0.91	4.31	0.88	0.00	0.88	3.71	0.57	0.27	0.41
5	4.32	0.91	0.00	0.91	4.50	1.00	0.00	1.00	4.71	1.00	0.00	1.00
6	4.36	0.77	0.01	0.77	4.31	0.75	0.03	0.74	4.57	1.00	0.00	1.00
7	4.27	0.73	0.02	0.72	4.13	0.69	0.07	0.67	4.71	0.86	0.05	0.85
8	3.68	0.55	0.15	0.47	4.06	0.75	0.03	0.74	4.57	1.00	0.00	1.00
9	4.27	0.82	0.00	0.82	4.25	0.88	0.00	0.88	3.86	0.86	0.05	0.85
10	4.45	0.82	0.00	0.82	4.44	0.88	0.00	0.88	4.86	1.00	0.00	1.00
11	4.41	0.82	0.00	0.82	4.31	0.88	0.00	0.88	4.43	0.86	0.05	0.85
12	4.32	0.95	0.00	0.95	4.50	1.00	0.00	1.00	4.86	1.00	0.00	1.00
13	4.50	1.00	0.00	1.00	4.44	0.94	0.00	0.94	4.57	1.00	0.00	1.00
14	4.41	0.82	0.00	0.82	4.19	0.75	0.03	0.74	3.71	0.57	0.27	0.41
15	4.36	0.86	0.00	0.86	4.38	0.88	0.00	0.88	4.71	1.00	0.00	1.00
16	3.95	0.73	0.02	0.72	4.13	0.81	0.01	0.81	4.43	1.00	0.00	1.00
17	4.18	0.77	0.01	0.77	4.13	0.81	0.01	0.81	4.29	0.86	0.05	0.85
18	4.41	0.77	0.01	0.77	4.25	0.75	0.03	0.74	4.86	1.00	0.00	1.00
19	4.45	0.82	0.00	0.82	4.44	0.88	0.00	0.88	4.71	1.00	0.00	1.00
20	4.00	0.68	0.04	0.67	4.06	0.75	0.03	0.74	4.86	1.00	0.00	1.00
21	4.18	0.82	0.00	0.82	4.06	0.88	0.00	0.88	4.86	1.00	0.00	1.00
22	4.36	0.91	0.00	0.91	4.50	1.00	0.00	1.00	4.71	1.00	0.00	1.00
23	4.45	0.91	0.00	0.91	4.25	0.81	0.01	0.81	4.86	1.00	0.00	1.00
24	4.32	0.77	0.01	0.77	4.00	0.75	0.03	0.74	4.29	0.86	0.05	0.85
25	4.36	0.95	0.00	0.95	4.13	0.88	0.00	0.88	4.71	1.00	0.00	1.00
26	4.41	0.86	0.00	0.86	4.44	0.94	0.00	0.94	5.00	1.00	0.00	1.00
27	4.41	0.86	0.00	0.86	4.38	0.81	0.01	0.81	4.71	0.86	0.05	0.85

I-CVI is the item-level content validity index. p_c_ is the probability of a chance occurrence. It was computed using the formula: p_c_ = 0.5^N^ [N!/A!(N-A)!], where *N* = number of judges and A = number agreeing on good relevance. k^*^ is kappa designating agreement on relevance. It was computed using the formula: k^*^ = (I-CVI – p_c_)/(1- p_c_). Evaluation criteria for kappa using guidelines by Cicchetti and Sparrow (1981) and Fleiss (1981): fair = k^*^ of 0.40–0.59; good = k^*^ of 0.60–0.74; and excellent = k^*^ > 0.74.

## Study 2: initial validation of the EATS

The purpose of study 2 was to initially investigate the factor structure, discriminant validity, and internal consistency reliability of the preliminary version of the EATS among a sample of Chinese athletes.

### Method of study 2

#### Participants

A sample of 478 Chinese athletes from 16 sports was invited to participate in this study. Excluding invalid questionnaires, data from 460 athletes were used for data analysis. All athletes had experiences in competing at national or international games at the moment of data collection. Basic information on the participants is presented in Table 1. DeVellis and Thorpe (2021) recommended an item–participant ratio between 1:5 and 1:10 for factor analysis. The initial EATS includes 27 items, which means that our sample size was sufficient for our analytical purposes. In addition, a *post-hoc* RMSEA-based power analysis was conducted to estimate the statistic power of analysis with our sample size using power4SEM^1^ (Wang and Rhemtulla, 2021). Specifically, power analysis for the RMSEA test for not-close fit was computed assuming a population RMSEA (H1) of 0.01 [suggested by Mac Callum et al. (2006)] to test the H0 of RMSEA ≥0.05 with our sample size of 460, various df values (of 1–6-factor EFA = 204–324; 27-item and 19-item 4-factor ESEM = 101–249) and an alpha level of 0.05. It was revealed that statistic powers of our analyses to reject not-close fit equaled 1, which further supports our sample size is sufficient to achieve convincing statistic power in our analyses.

#### Procedure

Coaches were contacted and were provided information about the study to obtain permission to access their athletes. After receiving their approval, informed consent was obtained from athletes before data collection. In addition, for those athletes who were younger than 18 years old, informed consent was obtained from their coaches who were asked to act *in loco parentis*. All athletes participated in this study voluntarily and completed an anonymous online version of the EATS after the daily training. They were reminded in the instructions of the online survey that all of the information they provided would be absolutely confidential, especially as their coaches would not be able to access their responses. It took about 5 min to complete the questionnaire.

#### Measure

A preliminary version of the EATS consists of 27 items and includes 3 subscales of behavioral engagement (10 items), cognitive engagement (8 items), and affective engagement (9 items). Athletes were requested to indicate to what extent they disagree or agree with each statement that related to the latest session of athletic training on a 5-point Likert scale ranging from 1 (strongly disagree) to 5 (strongly agree).

### Data analysis

SPSS version 22 was used to calculate the descriptive statistics of variables, and Mplus 7.31 was used to conduct exploratory factor analysis (EFA) and exploratory structural equation model (ESEM) to investigate the factor structure of the EATS. First, EFAs were conducted using a robust maximum likelihood estimator (MLR) of Mplus to compare one to six correlated latent factor structures of the EATS. As recommended by Marsh et al. (2009, 2014), the EFAs were estimated with an oblique geomin rotation. The number of factors that have an eigenvalue over one and a large bend in the scree plot indicates the potential reasonable factor structure of the EATS. Meanwhile, goodness-of-fit indices of different solutions were consulted with better goodness-of-fit indices, indicating a reasonable and desirable solution.

Furthermore, given that confirmatory factor analysis (CFA) has been criticized relying on highly restrictive independent cluster model, in which cross-loading of items on unintended factors in multidimensional instruments are forced to be zero (Asparouhov and Muthen, 2009), ESEM has been proposed to overcome the limitations of CFA because it integrates the principles of EFA within the CFA/SEM framework and provides a better representation of complex multidimensional structures of instrument (Asparouhov and Muthen, 2009). ESEM has been widely used in studies from various domains that aim to examine the factor structure and measurement invariance of the instrument (Appleton et al., 2016). Therefore, second, ESEM using the maximum-likelihood robust (MLR) estimator was conducted to reexamine the factor structure and the performance of the items of the EATS. The fit of the models using EFA and ESEM approaches was compared, and the performance of items was evaluated. Multi-fit indices were used to evaluate the adequacy of the model fit to the data in the present study, including the chi-square value, comparative fit index (CFI), Tucker–Lewis index (TLI), root mean square error of approximation (RMSEA) accompanied by its 90% confidence interval (CI), and standardized root mean square residual (SRMR). Due to no specific model-data fit recommendations for the ESEM are available, the commonly recommended criterion for independent cluster model CFA approach was adopted in this study for both EFA and ESEM analysis (Appleton et al., 2016). Specifically, the thresholds of >0.90, close to (or less than) 0.08, and up to 0.08 for the CFI and TLI, SRMR, and RMSEA indices, respectively, indicate an acceptable fit. CFI and TLI values exceeding 0.95, and SRMR and RMSEA close to (or less than) 0.08 and 0.06, respectively, represent a good fit (Hu and Bentler, 1999). In terms of interpreting the extracted factors, items with primary-loading less than 0.40 and/or cross-loading more than 0.32 were considered problematic (Tabachnick and Fidell, 2019) and should be considered to be removed. Cronbach’s alpha coefficient (α) and composite reliability (CR) were calculated to evaluate the internal consistency reliability of the EATS. The value of α > 0.70 (Nunnally and Bernstein, 1994) and CR > 0.70 (Raykov and Shrout, 2002) indicates acceptable internal consistency reliability.

### Results of study 2

#### Factor structure

Table 3 presents the eigenvalues and goodness-of-fit indices of different solutions of EFA. It was suggested that the 4-factor solution outperformed other solutions because the eigenvalues were larger than 1, and meanwhile, the solution demonstrated an acceptable model fit. Although the 5- and 6-factor solution demonstrated better model fit than that of the 4-factor solution, some eigenvalues of the two solutions were smaller than 1. In addition, inspection of the factor loadings of the items revealed difficulties in interpreting the results of the two solutions. The four components of the four-factor solution could represent behavioral engagement, cognitive engagement, positive affective engagement, and negative affective engagement. Therefore, in the following ESEM analysis, the four-factor solution was adopted.

**Table 3:** Eigenvalues and goodness-of-fit indices of different solutions.

Model	Eigenvalues	*χ* ^2^	df	CFI	TLI	RMSEA (90% CI)	SRMR
1-Factor EFA	11.489	2104.325	324	0.750	0.729	0.109 (0.105–0.114)	0.075
2-Factor EFA	2.227	1390.535	298	0.847	0.819	0.089 (0.085–0.094)	0.051
3-Factor EFA	1.478	906.670	273	0.911	0.886	0.071 (0.066–0.076)	0.039
4-Factor EFA	1.222	663.263	249	0.942	0.918	0.060 (0.055–0.066)	0.028
5-Factor EFA	0.908	522.793	226	0.958	0.935	0.053 (0.047–0.059)	0.024
6-Factor EFA	0.773	434.961	204	0.968	0.944	0.050 (0.043–0.056)	0.021

EFA, exploratory factor analysis; χ^2^, robust chi-square test of exact fit; df, degrees of freedom; CFI, comparative fit index; RMSEA, root mean square error of approximation; CI, confidence interval; SRMR, standardized root mean square residual.

Results of the 27-item 4-factor ESEM demonstrated an acceptable model fit, χ^2^ (249) = 521.703, *p* < 0.001; CFI = 0.945; TLI = 0.922; SRMR = 0.028; RMSEA = 0.049 (90% CI: 0.043–0.055). Inspection of the factor loadings of items revealed that the primary loading of 6 items (items 3, 5, 12, 13, 22, and 23) was lower than 0.4 and cross-loading of 2 items (items 16 and 24) larger than 0.32 (see Table 4). The four items (items 1, 4, 8, and 14) that were rated as fair by either of the three samples in content validity analysis in study 1 were found to function well and therefore retained. However, item 3 which was rated as fair by both athletes and coaches in content validity analysis in study 1 was found problematic. Therefore, the 8 problematic items (items 3, 5, 12, 13, 16, 22, 23 and 24) were removed from further analysis. Excluding these 8 items much improved the fit of the model to the data: χ^2^ (101) = 189.857, *p* < 0.001; CFI = 0.972; TLI = 0.953; SRMR = 0.023; RMSEA = 0.044 (90% CI: 0.034–0.053). Further examination of the factor loadings revealed that primary loadings ranged from 0.51 to 0.84 and cross-loadings were smaller than 0.32 ranging from −0.01 to 0.26, which suggested that the 19 items of the EATS functioned well (see Table 4).

**Table 4:** Descriptive statistics, standardized factor loadings, inter-factor correlations, composite reliability, and Cronbach’s alpha of the 27-item ESEM and 19-item ESEM in study 2.

Factor	Items	Mean	SD	27-item ESEM				19-item ESEM						
				Cog E (λ)	Beh E (λ)	PA E (λ)	NA E (λ)	Cog E (λ)	Beh E (λ)	PA E (λ)	NA E (λ)	δ	CR	α
Cog E					0.41	0.35	−0.26		0.39	0.34	−0.26		0.82	0.86
	Item 4	3.80	0.77	**0.57**	0.14	−0.01	0.02	**0.59**	0.11	−0.06	−0.04	0.60		
	Item 6	3.84	0.76	**0.52**	0.22	0.10	−0.16	**0.51**	0.21	0.11	−0.14	0.46		
	Item 7	3.81	0.75	**0.58**	0.11	0.18	−0.06	**0.66**	0.08	0.13	−0.08	0.40		
	Item 11	3.85	0.77	**0.49**	0.22	0.16	0.00	**0.59**	0.18	0.09	−0.06	0.45		
	*Item 13*	*3.77*	*0.83*	***0.34***	*0.14*	*0.29*	*−0.21*							
	Item 15	3.82	0.73	**0.62**	0.12	0.20	−0.02	**0.70**	0.07	0.13	−0.07	0.33		
	*Item 23*	*3.52*	*0.82*	***0.26***	*0.30*	*0.17*	*−0.29*							
	Item 26	3.88	0.76	**0.47**	0.04	0.28	−0.01	**0.52**	0.02	0.24	−0.03	0.57		
Beh E						0.46	−0.37			0.42	−0.32		0.83	0.87
	Item 2	3.31	0.89	0.09	**0.46**	0.02	−0.20	0.14	**0.43**	0.01	−0.22	0.62		
	*Item 3*	*2.62*	*0.95*	*−0.05*	***0.00***	*−0.11*	*0.44*							
	*Item 5*	*4.03*	*0.70*	*0.60*	***0.07***	*0.05*	*−0.22*							
	Item 9	3.30	0.81	−0.09	**0.83**	0.16	0.00	−0.04	**0.84**	0.13	−0.03	0.20		
	Item 10	3.33	0.83	0.01	**0.78**	0.18	0.00	0.09	**0.77**	0.14	−0.04	0.21		
	*Item 12*	*3.91*	*0.73*	*0.45*	***0.12***	*0.15*	*−0.31*							
	Item 14	3.47	0.90	0.17	**0.52**	0.14	−0.14	0.21	**0.49**	0.14	−0.14	0.45		
	Item 17	3.37	0.78	0.16	**0.58**	0.06	−0.11	0.19	**0.54**	0.05	−0.13	0.47		
	*Item 22*	*3.78*	*0.77*	*0.41*	***0.26***	*0.07*	*−0.17*							
	*Item 24*	*3.50*	*0.80*	*0.38*	***0.48***	*0.06*	*−0.18*							
PA E							−0.51				−0.50		0.85	0.89
	Item 18	4.29	1.63	−0.01	0.13	**0.76**	−0.12	0.05	0.15	**0.70**	−0.18	0.20		
	Item 19	4.02	1.62	0.04	0.03	**0.83**	−0.08	0.07	0.05	**0.81**	−0.10	0.17		
	Item 25	4.11	1.64	−0.09	0.16	**0.66**	−0.14	−0.05	0.19	**0.61**	−0.18	0.37		
	Item 27	4.25	1.60	0.11	0.01	**0.58**	−0.10	0.14	0.01	**0.55**	−0.13	0.56		
NA E													0.72	0.75
	Item 1	2.72	0.99	0.03	−0.11	−0.16	**0.51**	0.01	−0.13	−0.12	**0.52**	0.59		
	Item 8	2.53	0.96	−0.14	0.10	−0.03	**0.59**	−0.13	0.06	−0.02	**0.55**	0.75		
	*Item 16*	*3.36*	*0.96*	*0.36*	*−0.20*	*−0.02*	***0.41***							
	Item 20	2.89	0.99	0.13	−0.08	−0.21	**0.59**	0.05	−0.07	−0.10	**0.68**	0.48		
	Item 21	2.82	0.91	0.05	0.10	−0.21	**0.59**	0.01	0.07	−0.15	**0.62**	0.35		

λ, standardized factor loading; SD, standard deviation; Cog E, cognitive engagement; Beh E, behavioral engagement; PA E, positive affective engagement; NA E, negative affective engagement; δ, uniquenesses; CR, composite reliability; α, Cronbach’s alpha; Primary loadings are indicated in bold; inter-factor correlation coefficients are underlined. Problematic items are marked in italics.

#### Discriminant validity and internal consistency reliability

It was found that the inter-factor correlations (ESEM results) ranged from −0.50 to 0.42, which suggested that the four factors were distinctive but correlated with each other (Table 4). These results provided initial support for the discriminant validity of the 19-item EATS. All four subscales demonstrated acceptable internal consistency reliabilities with composite reliability (CR) ranging from 0.72 to 0.85 and Cronbach’s alpha coefficients ranging from 0.75 to 0.89. Table 4 displays item means, standard deviations, standardized factor loadings, inter-factor correlations, composite reliability, and Cronbach’s alpha coefficients of the EATS subscales.

## Study 3: further examination of the psychometric properties of the EATS

The purpose of study 3 was to further investigate the factor structure, discriminant validity, internal consistency reliability, and nomological validity of the EATS among an independent sample of Chinese athletes. Furthermore, measurement invariance of the EATS across samples and genders was examined.

### Method of study 3

#### Participants and procedure

A sample of 550 Chinese athletes from 11 sports were invited to participate in this study. Excluding invalid questionnaires, data from 513 athletes were used for data analysis. All athletes had experiences in competing at national or international games at the moment of data collection. Basic information on the participants is presented in Table 1. According to the results of the *post-hoc* RMSEA-based power analysis conducted in study 2, the sample size of 513 is sufficient for our statistical analyses. The procedure was the same as that in study 2. It took about 10 min to complete all questionnaires in study 3.

#### Measure

The 19-item EATS was used to measure athletes’ behavioral engagement (6 items), cognitive engagement (5 items), positive affective engagement (4 items), and negative affective engagement (4 items). Athletes were requested to indicate to what extent they disagreed or agreed with each statement that related to the latest session of athletic training on a 5-point Likert scale ranging from 1 (strongly disagree) to 5 (strongly agree) (see Supplementary material C).

A 5-item Chinese version of the Subjective Vitality Scale (SVS; Ryan and Frederick, 1997; Liu and Chung, 2019) was employed to measure athletes’ subjective vitality. Responses were provided on a 7-point Likert scale ranging from 1 (strongly disagree) to 7 (strongly agree). Previous research has suggested that the SVS displayed satisfactory validity and reliability among Chinese athletes (Liu et al., 2022). Internal consistency reliability of the scale in previous research (*α* = 0.954) and in the present study (*α* = 0.955) was satisfactory.

A 4-item Chinese version of the concentration disruption subscale of the Sport Anxiety Scale-2 (SAS-2; Smith et al., 2006) was used to measure participants’ concentration disruption. Responses were measured on a 4-point Likert scale ranging from 1 (never) to 4 (always). Previous research revealed that the concentration disruption subscale displayed satisfactory validity and reliability among Chinese elite athletes (Liu et al., 2022). Internal consistency reliability of the concentration disruption subscales in previous research (*α* = 0.896) and in the present study (*α* = 0.895) were satisfactory.

A 3-item Chinese version of the effort subscale of the Intrinsic Motivation Inventory (IMI; McAuley et al., 1989) was used to measure participants’ effort input in their athletic training. Responses were measured on a 7-point Likert scale ranging from 1 (completely disagree) to 7 (completely agree). Previous research revealed that the effort subscale displayed satisfactory validity and reliability among athletes (McAuley et al., 1989). Internal consistency reliability of the concentration disruption subscale in previous research (*α* = 0.84) was satisfactory. In the present study, the internal consistency reliability was slightly lower than 0.7 (*α* = 0.676).

A nine-item Chinese version of the International Positive and Negative Affect Schedule Short Form (I-PANAS-SF; Thompson, 2007; Liu et al., 2020) was used to measure athletes’ positive affect (four items) and negative affect (five items) in general in this study. Responses were provided on a 5-point Likert scale ranging from 1 (never) to 5 (always). Previous research revealed that the Chinese version of the I-PANAS-SF displayed satisfactory validity and reliability among Chinese athletes (e.g., Liu et al., 2022). Internal consistency reliability of the I-PANAS-SF in a previous study (*α* = 0.755–0.88) and the current study (*α* = 0.758–0.82) was satisfactory.

#### Data analysis

First, ESEM using an MLR estimator was conducted to further examine the factor structure of the 19-item EATS. Second, Cronbach’s alpha coefficients and composite reliability (CR) were calculated to evaluate the internal consistency reliability of the EATS. Third, the nomological validity of the EATS was evaluated by examining correlations between subscales of EATS with their theoretically related variables. Finally, measurement invariance of the EATS across samples (study 2 and study 3) and genders (male athletes from study 2 and study 3 vs. female athletes from study 2 and study 3) were investigated using a sequential model testing approach via multiple-group ESEM. Specifically, four models, namely, configural, metric invariance (weak invariance), scalar invariance (strong invariance), and item uniqueness invariance (strict invariance) were evaluated (Meredith, 1993). Due to the chi-square difference test being sensitive to the sample size, the differences in the descriptive fit indices (ΔCFI, ΔRMSEA, and ΔSRMR) were used in model comparisons in this study. According to Chen (2007), when testing metric invariance, non-invariance is indicated by a change of ≥ 0.010 in the CFI supplemented by a change of ≥ 0.015 in the RMSEA or ≥ 0.030 in the SRMR; when testing scalar or uniqueness invariance, non-invariance is indicated by a change of ≥ 0.010 in the CFI supplemented by a change of ≥ 0.015 in the RMSEA or ≥ 0.010 in the SRMR.

### Results of study 3

#### Factor structure, discriminant validity, and internal consistency reliability

The ESEM results suggested that the 19-item 4-factor ESEM displayed a good fit to the data, *χ*^2^ (101) = 191.43, *p* < 0.001, CFI = 0.975, TLI = 0.958, SRMR =0.021, RMSEA =0.042 (90% CI: 0.033–0.051). Primary loadings were larger than 0.4 (ranging from 0.44 to 0.81), and cross-loadings were smaller than 0.32 (ranging from −0.25 to 0.27), which suggested that the 19 items of the EATS functioned well. Low-to-moderate inter-factor correlations (−0.43 to 0.49) were revealed. These results suggested that the four factors of the EATS were distinctive from but related to each other, which provided further support for the discriminant validity of the EATS. All four subscales demonstrated acceptable internal consistency reliabilities with composite reliability (CR) ranging from 0.74 to 0.84 and Cronbach’s alpha coefficients ranging from 0.74 to 0.88. Table 5 displays item means, standard deviation, standardized factor loadings, inter-factor correlations, and internal consistency reliability of the EATS.

**Table 5:** Descriptive statistics, standardized factor loadings, inter-factor correlations, composite reliability, and Cronbach’s alpha of the 19-item ESEM in study 3.

Factor	Items	Mean	SD	19-item ESEM						
				Cog E (λ)	Beh E (λ)	PA E (λ)	NA E (λ)	δ	CR	α
Cog E					0.49	0.49	−0.23		0.81	0.87
	Item 4	3.90	1.06	**0.58**	−0.01	0.03	0.00	0.64		
	Item 6	4.21	0.93	**0.44**	0.24	0.20	−0.09	0.42		
	Item 7	4.14	0.93	**0.72**	0.06	0.12	−0.07	0.30		
	Item 11	4.11	0.93	**0.50**	0.24	0.12	−0.04	0.45		
	Item 15	4.12	0.91	**0.54**	0.27	0.12	−0.09	0.34		
	Item 26	4.06	1.00	**0.55**	0.01	0.23	−0.09	0.47		
Beh E						0.41	−0.20		0.84	0.88
	Item 2	3.63	1.13	0.21	**0.48**	0.09	−0.06	0.55		
	Item 9	3.72	1.05	0.15	**0.74**	0.04	−0.06	0.28		
	Item 10	3.80	1.07	0.06	**0.80**	0.09	−0.09	0.20		
	Item 14	3.93	1.03	0.11	**0.50**	0.25	−0.02	0.48		
	Item 17	3.58	1.03	0.07	**0.63**	0.18	−0.02	0.41		
Pos E							−0.43		0.83	0.88
	Item 18	3.96	1.13	0.14	0.04	**0.70**	−0.12	0.28		
	Item 19	4.04	1.11	0.02	0.06	**0.81**	−0.10	0.20		
	Item 25	3.89	1.11	0.11	0.03	**0.56**	−0.25	0.40		
	Item 27	3.92	1.12	0.13	0.15	**0.52**	−0.07	0.47		
Neg E									0.74	0.74
	Item 1	2.12	1.20	−0.08	−0.09	−0.04	**0.56**	0.60		
	Item 8	2.08	1.28	0.06	0.15	−0.13	**0.46**	0.75		
	Item 20	2.30	1.25	0.00	−0.11	−0.04	**0.67**	0.48		
	Item 21	2.19	1.24	0.00	0.04	−0.06	**0.79**	0.35		

λ, standardized factor loading; SD, standard deviation; Cog E, cognitive engagement; Beh E, behavioral engagement; PA E, positive affective engagement; NA E, negative affective engagement; δ, uniquenesses; CR, composite reliability; α, Cronbach’s alpha; Primary loadings are indicated in bold; inter-factor correlation coefficients are underlined.

#### Nomological validity

Previous research in work and academic settings has revealed that engagement is positively associated with vitality (Chughtai and Buckley, 2011; Bakker et al., 2020), subjective wellbeing (Ouweneel et al., 2012; Matthews et al., 2014; King et al., 2015; Garg and Singh, 2019), trait flow (Smith et al., 2023), and satisfaction (Alarcon and Lyons, 2011; Derbis et al., 2018) and negatively associated with burnout and negative affect (Bledow et al., 2011; Upadyaya et al., 2016; Chen et al., 2020). Based on this evidence, it is reasonable to assume that athletes’ behavioral engagement, cognitive engagement, and positive affective engagement would be positively associated with their subjective vitality, positive affect in general, and effort put into athletic training while negatively related to negative affect in general and concentration disruption in athletic training. On the other hand, we anticipated that athletes’ negative affective engagement would be negatively associated with the abovementioned positive outcomes (subjective vitality, positive affect in general, and effort put in athletic training) and negative outcomes (negative affect in general and attentional distraction in athletic training). Therefore, the nomological validity was evaluated by examining the relationships between EATS subscales with the abovementioned positive and negative outcomes. Table 6 displays the correlations among variables. As hypothesized, moderate-to-high positive correlations were evidenced among behavioral engagement, cognitive engagement, and positive affective engagement with positive outcomes. Meanwhile, moderate negative associations were found among behavioral engagement, cognitive engagement, and positive affective engagement with negative outcomes. In addition, negative affective engagement was found negatively associated with positive outcomes while positively associated with negative outcomes. These findings provided support for the nomological validity of the EATS.

**Table 6:** Correlations between EATS subscales with theoretical related variables in nomological validity.

Variables	α	Cog E	Beh E	PA E	NA E
SV	0.955	0.689**	0.633**	0.765**	−0.421**
PA	0.758	0.548**	0.494**	0.601**	−0.429**
Effort	0.676	0.535**	0.458**	0.432**	−0.452**
Distraction	0.896	−0.424**	−0.413**	−0.400**	0.506**
NA	0.820	−0.380**	−0.458**	−0.415**	0.468**

**p < 0.01; SV, subjective vitality; PA, positive affect; NA, negative affect; Cog E, cognitive engagement; Beh E, behavioral engagement; PA E, positive affective engagement; NA E, negative affective engagement; α, Cronbach’s alpha.

#### Invariance testing across samples and genders

Table 7 displays the goodness-of-fit indices for the invariance analysis of the EATS measurement model across samples and genders. For the invariance analysis across samples, there were no substantial changes in the selected indices (ΔCFI, ΔRMSEA, and ΔSRMR) for metric and scalar invariance tests. However, the changes in CFI and SRMR were larger than the cut-off values (ΔCFI = 0.034 > 0.01; ΔRMSEA = 0.015; ΔSRMR = 0.043 > 0.01) for the uniqueness invariance test. Therefore, we concluded that the weak and strong invariance of the EATS measurement model across samples was supported. For the invariance analysis across genders, there were no substantial changes in the selected indices (ΔCFI, ΔRMSEA, and ΔSRMR) for metric and scalar invariance tests. Although it was found that the change in SRMR was larger than the cutoff value (ΔSRMR = 0.013 > 0.01) for the uniqueness invariance test, no substantial changes were evidenced in the CFI and RMSEA (ΔCFI = 0.003 < 0.01; ΔRMSEA = 0.001 < 0.01). Therefore, we concluded that weak, strong, and strict invariance of the EATS measurement model across genders was supported.

**Table 7:** Measurement invariance across samples and genders.

EATS	Model	χ^2^	df	CFI	TLI	RMSEA (90%CI)	SRMR	ΔCFI	ΔRMSEA	ΔSRMR
Across samples	Configural	381.308	202	0.974	0.956	0.043 (0.036/0.049)	0.022	-	-	-
	Metric (weak invariance)	464.621	262	0.971	0.962	0.040 (0.034/0.046)	0.037	−0.003	−0.003	0.015
	Scalar (strong invariance)	498.795	277	0.968	0.960	0.041 (0.035/0.046)	0.040	−0.003	0.001	0.003
	Uniqueness (strict)	752.432	296	0.934	0.924	0.056 (0.051/0.061)	0.083	−0.034	0.015	0.043
Across genders	Configural	379.748	202	0.974	0.956	0.043 (0.036/0.049)	0.022	-	-	-
	Metric (weak invariance)	448.320	262	0.972	0.964	0.038 (0.032/0.044)	0.041	−0.002	−0.005	0.019
	Scalar (strong invariance)	479.383	277	0.970	0.963	0.039 (0.033/0.045)	0.042	−0.002	0.001	0.001
	Uniqueness (strict invariance)	521.905	296	0.967	0.961	0.040 (0.034/0.045)	0.055	−0.003	0.001	0.013

χ^2^, robust chi-square test of exact fit; df, degrees of freedom; CFI, comparative fit index; RMSEA, root mean square error of approximation; CI, confidence interval; SRMR, standardized root mean square residual; ΔCFI, changes in CFI; ΔRMSEA, changes in RMSEA; ΔSRMR, changes in SRMR.

## General discussion

In competitive sports, athlete engagement has been extensively examined, which is mainly related to athletes’ continuous and positive emotional and cognitive feelings toward their sports in general. However, it could not be used to reflect to what extent athletes have engaged in specific sessions of their athletic training and further their training quality. In contrast, athletes’ engagement in athletic training reflects the intensity of athletes’ productive involvement with their training-related activities and tasks and could be used to reflect athletes’ engagement in athletic training, which could be treated as one of the indicators to monitor the training quality of athletes. To the best of our knowledge, no psychometrically sound and context-specific measure of athletes’ engagement in athletic training has been developed yet. To address this deficit, in a series of three rigorous studies, we conceptualized the definition and structure of engagement in athletic training and further initially developed and evaluated the psychometric properties of the Engagement in Athletic Training Scale (EATS). Collectively, it was found that engagement in athletic training could be characterized as four distinct but related dimensions behavioral engagement, cognitive engagement, positive affective engagement, and negative affective engagement. Psychometric analyses revealed that the 19-item EATS displayed satisfactory reliability and validity and could be used to assess athletes’ engagement in athletic training. The current study adds new to the literature in terms of clarifying the concept and structure of engagement in athletic training and providing a valid and reliable instrument that could be used to assess athletes’ engagement in athletic training, which makes further investigations on the antecedents and consequences of engagement in athletic training possible.

The definition and structure of engagement in athletic training have not been clarified and investigated in previous research, which is the main reason that work on this particular topic was rare in the literature. In study 1, we conducted face-to-face semi-structured interviews with coaches and athletes and qualitatively analyzed the verbatim transcripts of interviews using content analysis. It was revealed that athletes’ engagement in athletic training could be represented by three distinct but correlated dimensions behavioral engagement, cognitive engagement, and affective engagement. This is consistent with previous research on student engagement (Nystrand and Gamoran, 1991; Ben-Eliyahu et al., 2018). We defined engagement in athletic training as the intensity of athletes’ productive involvement with their training-related activities and tasks. The operational definition was organized as the extent to which athletes execute, comprehend analyze, and affectively (both positively and negatively) engage in their training-related tasks. This definition reflects both overt behaviors and covert cognitive and affective activities throughout the process of athletic training. In practice, coaches usually evaluate the training quality of athletes by observing those over indicators including physical behaviors (quantity that athletes finish in athletic training such as repetitions, training intensity, and volume), physical movements (quality of the movements and actions such as accuracy and stability), and performance outcomes (concrete performance). However, it is impossible for coaches to directly observe to what extent the athletes have cognitively engaged in their athletic training. For example, one of the typical feedbacks from coaches is that some athletes only physically repeat the movements and actions without thinking about the rationales behind and requirements of the training tasks, which is the main reason that little or no improvements were observed after training for a period of time although they seemed having worked very hard in their athletic training. Similarly, it is difficult for coaches to accurately observe the affective states of athletes in the process of athletic training because most athletes are prone to hide their true emotions, especially those negative ones to avoid coaches judging them as mentally weak or not tough enough. Therefore, it is imperative to develop an instrument that could be used to assess athletes’ engagement in athletic training as a supplemental tool to monitor their training quality in addition to observations from coaches. To achieve this purpose, in study 1, we first developed a pool with 27 EATS items based on the results of qualitative analysis. Although it was revealed that there was some inconsistency across samples on the content validity of 4 (out of 27) items, we decided to retain all items to be further examined in the following statistical analysis in study 2.

The results of study 2 revealed that the EATS successfully captured the core components of behavioral, cognitive, and affective engagement in athletic training. However, it is noteworthy that the three-solution EFA model is inferior to the four-solution EFA model, in which the affective engagement was divided into positive affective engagement and negative affective engagement. Although this result was inconsistent with previous findings in academic engagement, it logically makes sense because both positive and negative affective engagements were reported by athletes and coaches, and more importantly, they contributed to the overall engagement in athletic training differently. Further analysis using the ESEM approach revealed that the 4-factor structure of the 19-item EATS demonstrated a good model fit to the data after removing the eight problematic items including items rated as fair in content validity analysis. Moderate inter-factor correlations among the four dimensions were revealed, which were consistent with previous findings and provided support for the discriminant validity. In other words, the four dimensions were distinctive from but associated with each other. For the negative affective engagement and positive affective engagement, a significant and negative correlation was revealed, which suggested that they may co-exist in one particular athletic training session. This result further reminds researchers and practitioners that athletic training is a dynamic and complex process, in which athletes’ affective states may change from moment to moment or across training tasks. In practice, some athletes always mention that they would be more physically involved in those activities they like rather than those they do not. The result further highlights that athletes’ engagement in athletic training would provide more detailed information that athlete engagement could not provide. For example, although an athlete may have reported high scores on athlete engagement (continuous and positive emotional and cognitive feelings toward their sports in general), she or he may also experience different affective engagements in the process of specific athletic training. This further justified the rationale of the current study that athlete engagement was insufficient to reflect or monitor training quality in comparison with engagement in athletic training.

The results of study 3 provided further support for the factor structure, discriminant validity, and internal consistency reliability of the EATS. Moreover, nomological validity was evidenced by revealing that the EATS subscales were significantly associated with theoretically related variables. Although no previous research has purposely explored these relationships in the context of a competitive sport, previous findings on moderate relationships of academic engagement and work engagement with similar variables justified the rationales of those examined variables in nomological validity. Finally, invariance analyses across samples and genders provided further psychometric evidence for the EATS. Measurement invariance is one of the fundamental psychometric properties of psychometrically sound scales. Weak invariance examines whether the change in each item score corresponds to the change in the factor score across groups. Strong invariance measures whether the values of the observed variables reflect the values of the latent variables in the same way across different groups. Strict invariance examines whether meaningful and unbiased comparisons can be made across groups and evidence of strict invariance means that any difference between groups is a true difference rather than a measurement artifact. The results of invariance analysis in study 3 revealed that weak and strong invariance of EATS across samples were supported while weak, strong, and strict invariance across genders were supported. Based on these results, we can conclude that athletes from different samples (from study 2 and study 3) interpreted the EATS items in a similar way (weak invariance) and the mean scores of participants from different samples on the EATS subscales were comparable (strong invariance). Similarly, for gender invariance analysis, it was concluded that female athletes and male athletes interpreted the EATS items in a similar way (weak invariance), and the mean scores reported by female athletes and male athletes on the EATS subscales were comparable (strong invariance), and moreover, if there were significant differences across genders, they were true and meaningful differences.

Collectively, the current study clarified and conceptualized the definition and structure of engagement in athletic training, contributing new theoretical insights to the literature on this topic. A psychometrically sound and context-specific instrument of the EATS was initially developed and validated among two independent samples, which displayed satisfactory validity and reliability. These results suggest that EATS could be used to assess athletes’ engagement in athletic training for both research and practical purposes. However, several limitations in this study should be acknowledged. First, a convenient sampling approach was used, although athletes from various sports were invited, athletes from many other sports (e.g., rowing and winter sports) were not invited in this study. The results of the current study may not be generalizable to athletes from other sports. Future researchers are encouraged to further investigate the psychometric properties of the EATS in more representative samples. Second, only athlete-level variance was considered; coach-level, sport-level, and institutional-level effects were not examined. Given that athletes are naturally nested in coaches, sports, and institutions, future studies are encouraged to further investigate the research question using multi-level design to differentiate effects at different levels purposely. Third, for the nomological validity, assumed theoretically related variables from other fields (work engagement and academic engagement) were used in this study, future studies may consider further identifying and investigating antecedences and consequences of the engagement in athletic training. Fourth, although measurement invariance analysis across samples and genders was examined in this study, given the distributions of age and sporting experience levels were unequal, the measurement invariance across these two groups was not examined. In addition, longitudinal invariance was not examined, which is important for longitudinal and interventional research. Future researchers are suggested to shed light on these research questions.

## Data availability statement

The raw data supporting the conclusions of this article will be made available by the authors, without undue reservation.

## Ethics statement

The studies involving humans were approved by Institutional Review Board at the Sun Yat-sen University. The studies were conducted in accordance with the local legislation and institutional requirements. Written informed consent for participation in this study was provided by the participants’ legal guardians/next of kin.

## Author contributions

J-DL: Conceptualization, Data curation, Formal analysis, Methodology, Project administration, Resources, Validation, Writing – original draft, Writing – review & editing. J-XW: Investigation, Methodology, Writing – original draft. Y-DZ: Investigation, Resources, Validation, Writing – review & editing. Z-HW: Data curation, Formal analysis, Writing – original draft, Writing – review & editing. SZ: Methodology, Writing – review & editing. J-CH: Data curation, Formal analysis, Project administration, Writing – review & editing. HL: Conceptualization, Validation, Writing – original draft, Writing – review & editing.

## Footnotes

^1^https://sjak.shinyapps.io/power4SEM/
