# Supplementary material for: Development and initial validation of the Engagement in Athletic Training Scale
Source: Front Psychol. 2024 Jul 23;15:1402065. doi: 10.3389/fpsyg.2024.1402065 (PMC11301410; doi:10.3389/fpsyg.2024.1402065)
Supplement: Supplementary file 1 [file Data_Sheet_1.PDF]

Supplementary A: Interview Guidelines

Interview Guideline: Coach version	Interview Guideline: Athlete version
1. In the process of athletes' daily training (technical training, physical training, actual combat confrontation training, etc.), how do you evaluate theirs training quality (different training tasks, the evaluation of coaches may be different. The coach can answer the question on a case-by-case basis).<br>2. You have just mentioned many different aspects. Could you please further explain why would you evaluate athletes' daily training from these aspects? For each aspect, what exactly have you specifically evaluated in the process of athletes' training? Why are these aspects important?<br>3. Whether did your athletes know or not what aspects that you would usually use to evaluate their training input? If they knew, have you explicitly mentioned them to your athletes, or have they felt them?<br>4. If they did not know, what reasons do you think might be? In what ways do you think athletes usually evaluate their training process? Why?	1. In your daily training, how do you usually evaluate your training quality (technical training, physical training, actual combat confrontation training, etc.)? (different training tasks, athletes may have different evaluation angles. Athletes can answer the question on a case-by-case basis.)<br>2. You have just mentioned many different aspects. Could you please further explain why did you evaluate your training from these aspects? For each aspect, what exactly have you specifically evaluated? Why are these aspects important for you?<br>3. How did you develop your ways to evaluate your training? Was it taught by the coach or you set up them by himself?<br>4. In what ways do you think your coaches usually evaluate the quality of your training? Why did your coach evaluate in that way?<br>5. How do you comment on the way your coach evaluating your training process? Can you evaluate your training accurately? Which aspects are usually more accurate and which may not be so accurate? What could be the reasons for the inaccuracy do you think?

Supplementary B: 27-ITEM EATS

运动员投入量表分量表及条目	EATS and subscales
**认知投入**	**Cognitive Engagement**
4、我在训练中会经常觉察自己的状态。	4. I am often aware of my mental and physical statues during the training.
6、我对训练任务的目的了解很清晰。	6. I know exactly the purpose of my training tasks.
7、我在训练中反复体会分析技术动作。	7. I keep practicing and analyzing my technical moves during the training.
11、我在训练中摸索适合自己的方式。	11. I always try to figure out ways that work for me during the training.
13、我理解教练所布置训练任务的理由。	13. I understand the reasons of the training tasks. *
15、我在训练过程中仔细思考技战术动作。	15. I keep thinking about my technical moves and tactics needed during the training.
23、我在训练过程中注意非常集中。	23. I am focus on the training tasks during the training. *
26、我在训练间歇会观察和分析别人的动作。	26. I observe and analyze technical moves of my teammates in breaks during the training.
**行为投入**	**Behavioral Engagement**
2、我达到了自己给自己定下的训练要求。	2. I achieved the training requirements assigned by myself.
3、我在训练中会找机会偷懒。	3. I look for opportunities to slack off in training. *
5、我会努力完成教练布置的任务。	5. I try my best to complete the training tasks arranged by coach. *
9、我达到了教练布置的技术要求。	9. I achieved the technical requirements set by my coach.
10、我很好的完成了教练布置的训练任务。	10. I successfully completed the training tasks assigned by my coach.
12、我积极配合教练的安排。	12. I am very collaborative during the training. *
14、我达到了教练布置的耐力要求。	14. I met the endurance requirements set by my coach
17、我达到了教练布置的难度要求。	17. I met the difficulty requirements set by my coach

22、我按照训练计划完成了训练任务。	22. I finished my training tasks as planned. *
24、我达到了教练布置的强度要求。	24. I met the intensity requirements set by my coach. *
**积极情感**	**Positive Affective Engagement**
18、我很享受训练过程。	18. I enjoy the training process.
19、我期待后续的训练。	19. I look forward to the next training session.
25、我觉得训练任务和内容有趣。	25. I think the training is very interesting.
27、训练时我感觉时间过的很快。	27. I feel time flies during the training.
**消极情感**	**Negative Affective Engagement**
1、我在训练过程中兴奋不起来。	1. I can't get excited during the training.
8、我对训练任务和内容很抵触。	8. I am very resistant to the training tasks and content.
16、我在训练过程中有些急躁。.	16. I lost my patience during the training. *
20、我在训练过程中情绪低落。	20. I feel low during the training.
21、我觉得训练任务和内容乏味。.	21. I feel the training tasks and content boring

Note. Items with * were removed based on the results of study 1 and study 2, which resulted in the 19-item version of EATS.

Supplementary C: 19-ITEM EATS

19-item No.	27-item No.		非常不同意 Strongly disagree				非常同意 Strongly agree
1	1	我在训练过程中兴奋不起来<br>I can't get excited during the training.	1	2	3	4	5
2	2	我达到了自己给自己定下的训练要求<br>I achieved the training requirements assigned by myself.	1	2	3	4	5
3	4	我在训练中会经常觉察自己的状态<br>I am often aware of my mental and physical statues during the training	1	2	3	4	5
4	6	我对训练任务的目的了解很清晰<br>I know exactly the purpose of my training tasks.	1	2	3	4	5
5	7	我在训练中反复体会分析技术动作<br>I keep practicing and analyzing my technical moves during the training.	1	2	3	4	5
6	8	我对训练任务和内容很抵触<br>I am very resistant to the training tasks and content.	1	2	3	4	5

7	9	我达到了教练布置的技术要求<br>I achieved the technical requirements set by my coach.	1	2	3	4	5
8	10	我很好的完成了教练布置的训练任务<br>I successfully completed the training tasks assigned by my coach.	1	2	3	4	5
9	11	我在训练中摸索适合自己的方式<br>I always try to figure out ways that work for me during the training.	1	2	3	4	5
10	14	我达到了教练布置的耐力要求<br>I met the endurance requirements set by my coach	1	2	3	4	5
11	15	我在训练过程中仔细思考技战术动作<br>I keep thinking about my technical moves and tactics needed during the training.	1	2	3	4	5
12	18	我很享受训练过程<br>I enjoy the training process.	1	2	3	4	5
13	19	我期待后续的训练<br>I look forward to the next training session	1	2	3	4	5
14	20	我在训练过程中情绪低落<br>I feel low during the training.	1	2	3	4	5
15	21	我觉得训练任务和内容乏味<br>I feel the training tasks and content boring	1	2	3	4	5
16	17	我达到了教练布置的难度要求<br>I met the difficulty requirements set by my coach	1	2	3	4	5

17	25	我觉得训练任务和内容有趣<br>I think the training is very interesting.	1	2	3	4	5
18	26	我在训练间歇会观察和分析别人的动作<br>I observe and analyze technical moves of my teammates in breaks during the training.	1	2	3	4	5
19	27	训练时我感觉时间过的很快<br>I feel time flies during the training.	1	2	3	4	5
